# RETRACTED ARTICLE: The role of temperature on the global spread of COVID-19 and urgent solutions

**DOI:** 10.1007/s13762-020-02991-8

**Published:** 2020-11-19

**Authors:** I. Roy

**Affiliations:** University College London (UCL), IRDR, London, UK

The Editor-in-Chief has retracted this article. After publication, concerns were raised regarding the data analysis and proposed solutions. Independent post-publication peer review has concluded that the statistical analysis used to support the conclusions is not appropriate and the recommendations presented are not supported by the data and may not be safe. In order to protect public health the content has been removed. The author does not agree with this retraction.

